# *In situ* formation of silver nanoparticles in linear and branched polyelectrolyte matrices using various reducing agents

**DOI:** 10.1186/1556-276X-9-164

**Published:** 2014-04-04

**Authors:** Vasyl Chumachenko, Nataliya Kutsevol, Michel Rawiso, Marc Schmutz, Christian Blanck

**Affiliations:** 1 Department of Chemistry, Kiev Taras Shevchenko National University, 60 Volodymyrska, Kiev UA-01033, Ukraine; 2 Institut Charles Sadron (CNRS-UdS), 23 rue du Loess-BP 84047, 67034 Strasbourg, Cedex 2, France

**Keywords:** Branched polymers, Polyelectrolytes, Dextran, Polyacrylamide, Grafted copolymers, Hydrolysis, Silver nanoparticles

## Abstract

Silver nanoparticles were synthesized in linear and branched polyelectrolyte matrices using different reductants and distinct synthesis conditions. The effect of the host hydrolyzed linear polyacrylamide and star-like copolymers dextran-graft-polyacrylamide of various compactness, the nature of the reductant, and temperature were studied on *in situ* synthesis of silver sols. The related nanosystems were analyzed by high-resolution transmission electron microscopy and UV-vis absorption spectrophotometry. It was established that the internal structure of the polymer matrix as well as the nature of the reductant determines the process of the silver nanoparticle formation. Specifically, the branched polymer matrices were much more efficient than the linear ones for stable nanosystem preparation.

## Background

During the last decade, silver nanoparticles (Ag NPs) attract significant attention due to their unique optical, thermal, and electrical properties as well as their use as antibiotic materials, photocatalysts, and conductive nano-inks [1-7]. The methods to obtain Ag NPs of well-defined morphology, size, orientation, and complex pattern are the subject of numerous researches. In principle, physical and chemical techniques for nanometer-sized metal particle preparation can be used [7-12]. Such methods as chemical vapor deposition, chemical reduction, photolytic reduction, and radiolytic reduction are among them. Reduction of metal ions into neutral clusters is a commonly used treatment in chemical synthesis.

The high reactivity of Ag NPs raises difficulties in developing stable colloidal dispersions, since Ag NPs rapidly undergo agglomeration. Therefore, it is urgent to search the methods allowing the acquisition of nanosystems with high storage stability. Silver colloids stabilized by polymers in various solvents are extensively investigated by considering the linear and star-shaped polymers, polymer brushes, block copolymers, and even dendrimers [13-19]. However, the advantages of branched polymer matrices in comparison with their linear polymer analogs for *in situ* nanoparticles formation are still not clear. Yet, this knowledge is needed to prove or disprove the necessity of using expensive materials. The chemical nature of the polymer matrices, the nature of the reductant, and temperature affect the shape and the size of the particles [20-25]. The internal structure of the polymers could also influence the process of nanoparticle formation. The branched polymer architecture demonstrates an improvement in the ordering phenomenon. That is why such systems can differ in functionalities from their linear analogs. In the present paper, we have focused on the study of Ag sols synthesized *in situ* in linear and branched polyelectrolyte polymer matrices. The effect of reductant and temperature was discussed too.

## Methods

### Materials

Dextran with *M*_*w*_ = 7 × 10^4^ g mol^−1^ (referred as D70 throughout) was purchased from Sigma Aldrich, St Quentin Fallavier, France. Cerium (IV) ammonium nitrate (Sigma Aldrich, St Quentin Fallavier, France) was used as initiator of radical graft polymerization. Dextran samples and the cerium salt were used without further purification. Acrylamide (Sigma Aldrich, St Quentin Fallavier, France) was twice re-crystallized from chloroform and dried under vacuum at room temperature for 24 h. NaOH from Aldrich was used for alkaline hydrolysis of polymer samples. Sodium borohydride and hydrazine hydrate (Sigma Aldrich, St. Quentin Fallavier, France) were used for chemical reduction of silver nitrate in polymer solutions in order to synthesize Ag NPs.

### Polymer matrices

Branched copolymers were obtained by grafting polyacrylamide (PAA) chains onto dextran (D70) backbone [26]. The synthesis was carried out using a ‘grafting from’ method. The theoretical number of grafting sites per polysaccharide backbone depends on the ratio of Ce (IV) concentration to dextran one $\left(n=\frac{\left[\mathrm{molCe}\left(\mathrm{IV}\right)\right]}{\left[\mathrm{molDextran}\right]}\right)$. Thus, *n* was equal to 5 or 20, and the related dextran-graft-polyacrylamide copolymers were referred as D70-*g*-PAA5 and D70-*g*-PAA20. The linear PAA (*M*_*w*_ = 1.40 × 10^6^ g mol^−1^) was synthesized by radical polymerization. All polymers were characterized by size-exclusion chromatography (SEC).

The D70-*g*-PAA copolymers and linear PAA were saponified by alkaline hydrolysis using NaOH to obtain polyelectrolyte samples. The hydrolysis for all samples was carried out as follows: 2 g of D70-*g*-PAA (or PAA) was dissolved in 200 mL of water and then 10 mL of a 5-M NaOH aqueous solution was added. The mixture was placed in a water bath at 50°С. The probes were taken in 30 min and precipitated by acetone. All samples were freeze-dried after precipitation and kept under vacuum.

### *In situ* synthesis of Ag NPs in linear and branched polyelectrolytes matrices

Sodium borohydride and hydrazine hydrate were used for the chemical reduction of silver nitrate dissolved in polymer solutions. This reaction led to Ag NP formation. The ratio of Ag^+^ ions to acrylamide monomers was 1:3.

A 0.1-M silver nitrate solution was added to a polymer solution under active stirring and was kept at such conditions during 20 min for equilibrium achievement. Then, 0.1 M of sodium borohydride or 3.8 g L^−1^ hydrazine hydrate aqueous solutions were added and stirred for 20 min. The chemical reduction was conducted at 20°C, 40°C, 60°C, and 80°C. The solution turned dark reddish brown immediately after adding the reductant, which indicated the Ag NP formation.

### Size-exclusion chromatography

SEC analysis was carried out by using a multi-detection device consisting of a LC-10 AD Shimadzu pump (throughput 0.5 mL min^−1^; Nakagyo-ku, Kyoto, Japan), an automatic injector WISP 717+ from Waters (Milford, MA, USA), three coupled 30-cm Shodex OH-pak columns (803HQ, 804HQ, and 806HQ; Munich, Germany), a multi-angle light scattering detector DAWN F from Wyatt Technology (Dernbach, Germany), and a differential refractometer R410 from Waters. Distilled water containing 0.1 M NaNO_3_ was used as eluent. Dilute polymer solutions (*c* = 3 g L^−1^ < c* = 1 / [*η*]) were prepared, allowing for neglect of intermolecular correlations in the analysis of light scattering measurements.

### Potentiometric titration

Potentiometric titration of polyelectrolyte samples was performed using a pH meter pH-340 (Econix Express, St. Petersburg, Russia). HСl (0.2 N) and NaOH (0.2 N) were used as titrants. Polymer concentration was 2 g L^−1^. The polymer solutions were titrated with HCl up to pH 2 and then with NaOH up to pH 12. Previously, a fine blank titration (titration of non-hydrolyzed polymer) was made. The absorption of OH^−^ anions was calculated through the analysis of the titration curves and then the limits of these values were used to determine the conversion degree (*А*) of amide groups into carboxylate ones. All measurements were performed at *T* = 25.0°C under nitrogen.

### Viscosimetry

Viscosity measurements were performed at 25.0°C ± 0.1°C using an Ostwald-type viscometer. All polymers were dissolved in distilled water without added salt. The pH of the polyelectrolyte solutions were in the range 7.8 < pH < 8.2.

### Transmission electron microscopy

The identification of Ag NPs and their size analysis were carried out using high-resolution transmission electron microscopy (TEM). A Philips CM 12 (Amsterdam, Netherlands) microscope with an acceleration voltage of 120 kV was used. The samples were prepared by spraying silver sols onto carbon-coated copper grids and then analyzed.

### UV-vis spectroscopy

UV-vis spectra of silver sols were recorded by Varian Cary 50 scan UV-visible spectrophotometer (Palo Alto, CA, USA) in the range from 190 to 1,100 nm (in 2-nm intervals). Original silver sols were diluted 50 times before spectral measurements.

## Results and discussion

The main molecular characteristics of linear and branched polymers are reported in Table 1. Dextran content in D70-*g*-PAA5 and D70-*g*-PAA20 copolymers is less than 5%, suggesting that copolymers actually form star-like polymers with a dextran core and PAA arms [26]. Surprisingly, the values of the *z*-average radius of gyration, *R*_*z*_, are almost identical for both branched D70-PAA20 polymers and linear PAA macromolecules of equivalent molecular weights. As the star-like structure of the copolymers should rather yield a smaller radius of gyration, one may surmise that PAA-grafted chains are more extended in the D-*g*-PAA copolymers than usual. This assumption received support that is described in detail in [26]. As it was reported [26,27], the average conformation of grafted PAA chains is controlled by the grafting ratio: for D70-*g*-PAA20, it is close to that of a worm-like chain; for D70-*g*-PAA5, it differs from that of a worm-like chain, although it is definitely not random, namely, the PAA-grafted chains are highly extended near their tethering point and recover a random conformation far from this point. The number of grafted chains and their average conformation are closely related to the compactness of the branched macromolecules which can be assessed through the ratio *R*_*z*_^*2*^/*M*_*w*_[27] (see Table 1). When the ratio *R*_*z*_^*2*^/*M*_*w*_ is lower, the compactness is higher.

**Table 1:** **Molecular parameters of the D70- *g *-PAA copolymers and the linear PAA**

**Sample**	***M***_***w ***_**(×10**^**−6**^ **g mol**^**−1**^**)**	***R***_***z ***_**(nm)**	***R***_***z***_^**2**^**/*M***_***w ***_**(×10**^**3**^**)**	**Dextran content (weight%)**
D70-*g*-PАА5	2.15	85	3.36	3.26
D70-*g*-PАА20	1.43	64	2.87	4.89
PAA	1.40	68	3.23	-

The compactness becomes higher as the grafting ratio of the D70-*g*-PAA samples increases. However, for D70-*g*-PAA5 copolymers, this characteristic is close to that of linear PAA macromolecules (Table 1).

Star-like D-*g*-PAA copolymers and linear PAA were transformed into polyelectrolytes. During hydrolysis, some amide groups of the PAA chains were converted into carboxylate ones:

Alkaline hydrolysis of D70-*g*-PAA were not attended by irrelevant processes (breaking or cross-linking of macromolecules) as it was confirmed by SEC analysis of source and saponified samples.

In comparison with linear polyacrylamide, all branched polymers reveal higher values of conversion to anionic form due to compactness of their molecular structure in comparison with linear polymer. It leads to a higher local concentration of functional groups for non-linear polymer molecule (Table 2).

**Table 2:** Conversion degree of polymers (hydrolysis time 30 min)

**Sample**	***А *** **(%)**
D70-*g*-PAA5	35
D70-*g*-PAA20	37
PAA	28

**Figure 1:**
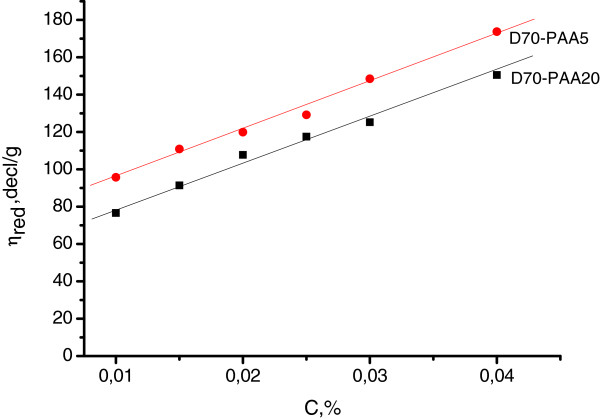
**Concentration dependence of reduced viscosity for hydrolyzed D70-*g*-PAA5 and D70-*g*-PAA20 samples.** Time of hydrolysis is 30 min.

The viscometry data reveals no polyelectolyte effect but a drastic increase in the intrinsic viscosity for hydrolyzed branched samples with respect to non-ionic ones (Figure 1). It is known that the reduced viscosity of polyelectrolyte solution increases in very dilute regime due to electrostatic repulsions between charged monomers. As it was mentioned above, grafted chains in D70-*g*-PAA copolymers, even in non-ionic form, have a worm-like or mushroom average conformation that is far from that of a random coil. Hydrolyzed D70-*g*-PAA copolymer in a salt form acquired limited extended conformation due to appearance of charged functional group. Therefore, its conformation cannot be changed when the concentration is decreased.

### NaBH_4_ as reducing agent

**Figure 2:**
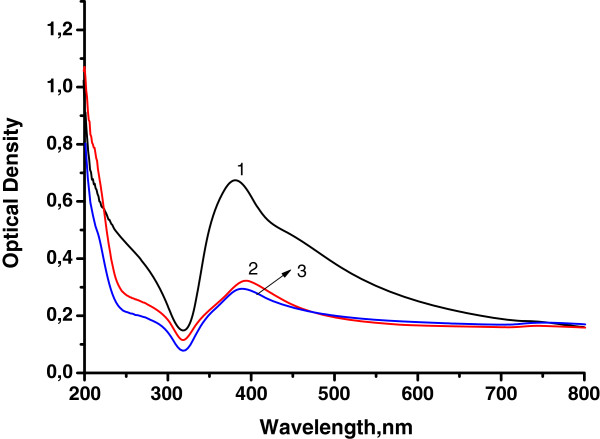
**UV-vis absorption spectra of silver sols synthesized in the polymer matrices.** D70-*g*-PAA20 (1), D70-*g*-PAA5 (2), and PAA (3). *T* = 20 C. The reductant is borohydride.

**Figure 3:**
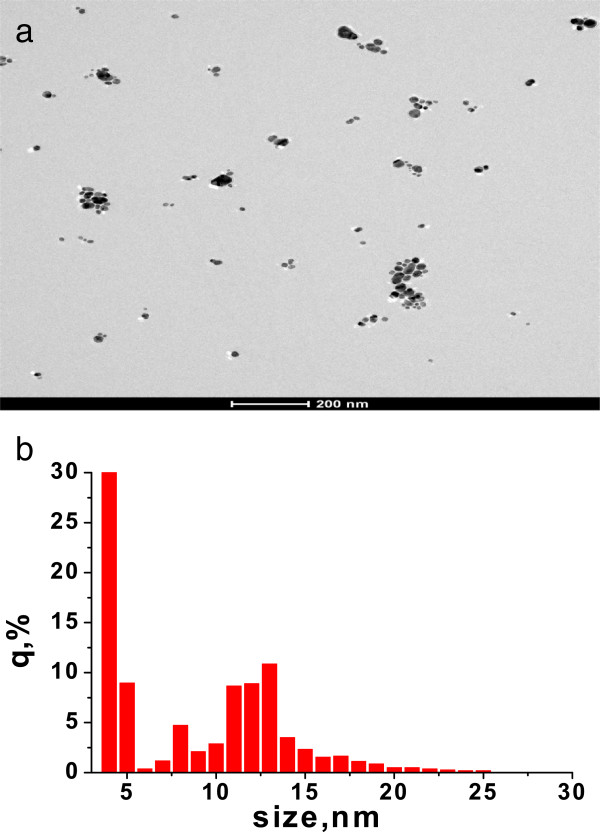
**TEM image (a) and nanoparticle size distribution (b) in silver sols synthesized in D70-PAA5 matrix.** The reductant is sodium borohydride.

The copolymers in anionic form were used as matrices for AgNP synthesis. Plasmon resonance absorption for all silver sols was observed at UV-vis spectra (Figure 2). The shoulder at first higher energy maximum in the range 275 to 282 nm may correspond to both small particles of 2 to 4 nm and Ag^+^ ions. The second maximum is situated at 390 to 410 nm; it corresponds to the plasmon absorption of Ag particles of 10 to 15 nm in size. Maximum intensity depends on polymer matrix type. The most efficient matrix for nanoparticle preparation is D70-*g*-PAA20 with the most compact internal structure (Table 1). The matrices of PAA and D70-*g*-PAA which are close in compactness reveal similar efficiency for nanoparticle synthesis. The shoulders in the plasmon peaks (Figure 2) imply that the synthesized sols contain either polydisperse nanoparticles with a significant fraction of aggregates for sols synthesized in linear PAA matrices or a high rate of small particles for nanosystems prepared in branched polymer matrices. Such conclusion was proved by TEM image analysis of silver sols. The TEM image and a size histogram are presented in Figure 3. Two types of particles are observed for sols synthesized in polyelectrolyte matrices (Figure 3). The first fraction corresponds to small spherical particles of 3 to 4 nm in size; the second one displays aggregated granules and spherical particles of 10 to 15 nm in size. Ag NPs synthesized in polyelectrolyte matrices differ from those prepared in non-ionic branched or linear matrices described previously [28,29]. It was shown that in non-ionic matrices, only spherical particles of 10 to 15 nm in size were formed. The bimodal size distribution of nanoparticles synthesized in polyelectrolyte matrices can be explained by the existence of two types of functional groups in the hydrolyzed macromolecules: amide and carboxylate ones. That can lead to two types of bonding with silver ions and provides two mechanisms of Ag NP formation.

**Figure 4:**
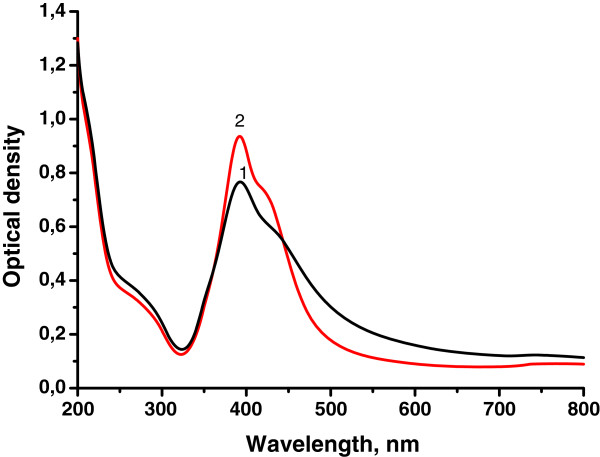
**UV-vis absorption spectra of silver sols synthesized in the polymer matrices.** D70-g-PAA20. (1) *T* = 40°C, (2) *T* = 60°C.

The effect of temperature on the process of silver sol formation is demonstrated in Figure 4. Highly concentrated stable sols were obtained using all branched polyelectrolytes as host polymers. An increase of temperature caused further Ag NP aggregation. This is revealed in the appearance of a shoulder of the resonance peak at 420 to 440 nm (Figure 4). Stable Ag sols could not be synthesized in linear PAA matrix. We observed the appearance of some precipitate at 40°C and 60°C. The phase separation occurred immediately at 80°C, while colloids synthesized in branched matrices remained stable.

### Hydrazine as reducing agent

**Figure 5:**
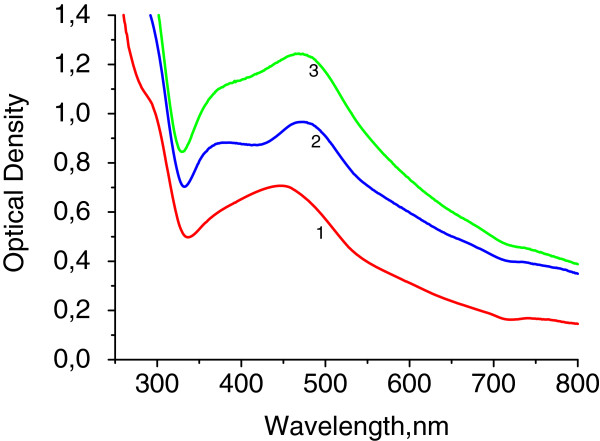
**UV-vis absorption spectra of silver sols synthesized in the polymer matrices.** D70-*g*-PAA20 (1), D70-*g*-PAA5 (2), and PAA (3). *T* = 20°C. The reductant is hydrazine hydrate.

**Figure 6:**
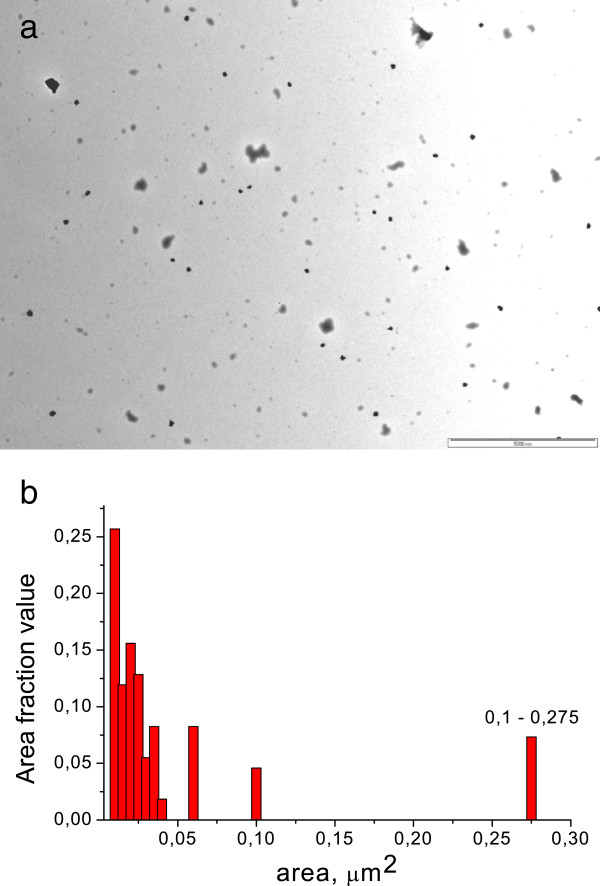
**TEM image (a) and area of nanoparticle distribution (b) in silver sols synthesized in D70-PAA5 matrix.** The reductant is hydrazine hydrate.

**Figure 7:**
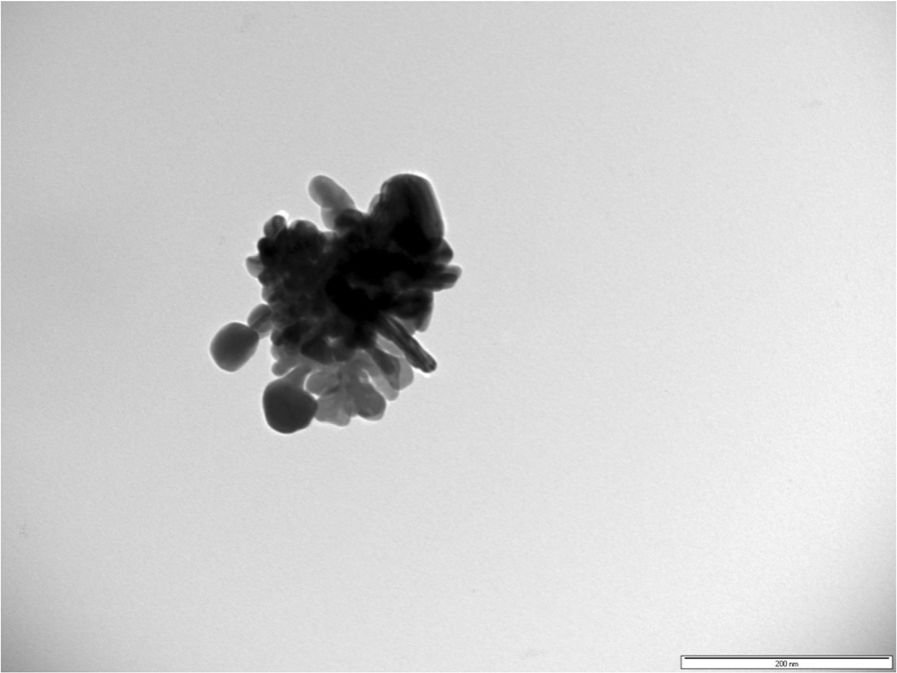
**TEM image of a single multi-branched silver particle.** The reductant is hydrazine hydrate.

Ag sols, obtained using hydrazine hydrate as reductant, display intensive plasmon absorption bands for all nanosystems synthesized in linear and branched polyelectrolyte matrices (Figure 5). For linear PAA, only one broad peak was registered in the range from 365 to 475 nm. Existence of two well-dedicated maxima for sols prepared in branched polymer matrices can be referred to different size fractions or to plasmon absorption of particles with anisotropic form. Both statements were proved by analysis of TEM images of silver sols (Figure 6a). Nanosystems were polydisperse (area distribution histogram is shown in Figure 6b), and single particles with average size of 130 ± 10 nm have anisotropic form. Large-scaled TEM revealed the presence of multi-branched Ag particles (Figure 7). Formation of hyperbranched anisotropic Ag nanostructures in aqueous solution was quite surprising; it is known that silver has a highly symmetric crystal structure. Similar anisotropic structures of Ag particles were described in [30-32]. It was concluded that hyperbranched structures result from slow-reducing nature (kinetically controlled growth) and shape-directing role of citric acid as reductant. In our case, the control of the Ag particle shape is realized also by the peculiarities of the host branched polymer internal structure. The most efficient matrix was D70-*g*-PAA20, i.e., the one formed by the macromolecules having the highest compactness (Table 1).

## Conclusions

The present study presents a study of Ag sols obtained in linear and branched polyelectrolyte matrices. It was revealed the effect of the internal structure of host polymer matrices depended on silver nanoparticle size, morphology, and stability. The polyelectrolyte linear polymer matrices were less efficient for silver sol manufacturing in comparison with branched ones for all reductants used. Something already contemplated and demonstrated for silver sol, synthesized *in situ* in the same polymer matrices using ascorbic acid as the reducing agent [33]. It was established that the temperature of synthesis and the reductant choice drastically affect the size and shape of silver nanoparticles obtained. Stable Ag sols could not be synthesized in linear PAA matrix at 80°C, while colloids synthesized in branched matrices remained stable.

## Competing interests

The authors declare that they have no competing interests.

## Authors’ contributions

VC and NK carried out the polymer and nanoparticle synthesis, polymer characterization, plasmon absorption study, and statistical analysis. MR carried out the SEC measurements and participated in the design of study and coordination. MS and CB carried out the TEM experiment. All authors read and approved the final manuscript.

## Authors’ information

VC is a Ph.D. student in the Macromolecular Department of Kiev Taras Shevchenko National University. NK is the principal researcher and is a Ph.D. and Dr. Chemical Science degree holder. MR is and Ph.D. and Dr. of Research degree holder and the head of team ‘Polyelectrolytes Complexes and Materials’. MS is a research engineer. CB is an engineer assistant.
